# Utilizing 5′ UTR Engineering Enables Fine-Tuning of Multiple Genes within Operons to Balance Metabolic Flux in *Bacillus subtilis*

**DOI:** 10.3390/biology13040277

**Published:** 2024-04-19

**Authors:** Jiajia You, Yifan Wang, Kang Wang, Yuxuan Du, Xiaoling Zhang, Xian Zhang, Taowei Yang, Xuewei Pan, Zhiming Rao

**Affiliations:** 1 Key Laboratory of Industrial Biotechnology of the Ministry of Education, Laboratory of Applied Microorganisms and Metabolic Engineering, School of Biotechnology, Jiangnan University, Wuxi 214122, China; youjiajia@jiangnan.edu.cn (J.Y.); wk15216426605@outlook.com (K.W.); jndx-duyuxuan@foxmail.com (Y.D.); 6220201057@stu.jiangnan.edu.cn (X.Z.); zx@jiangnan.edu.cn (X.Z.); yangtw@jiangnan.edu.cn (T.Y.); 2 Yixing Institute of Food and Biotechnology Co., Ltd., Yixing 214200, China; 3 Department of Food Science and Technology, Texas A & M University, College Station, TX 77843, USA; wangyifan@tamu.edu

**Keywords:** 5′-untranslated region (5′ UTR), synthetic operon, synthetic biology, riboflavin, *Bacillus subtilis*

## Abstract

**Simple Summary:**

The synthesis of natural or non-natural products in microorganisms frequently involves multiple genes or pathways. Enhancing and balancing the expression of these genes is essential to prevent growth inhibition and product synthesis blockage resulting from the accumulation of intermediate products, thereby effectively increasing the yield of target products. However, there is a deficiency in robust tools for balancing gene expression in *Bacillus subtilis*. Herein, we identified a 5′ UTR sequence that enhances gene expression levels. The library constructed from this sequence enables a broad spectrum of gene variations. Utilizing this library to construct a synthesized *rib* operon markedly boosts riboflavin production. Therefore, a novel 5′ UTR sequence was screened and characterized, supplementing the synthetic biology toolkit for *B. subtilis*.

**Abstract:**

The application of synthetic biology tools to modulate gene expression to increase yield has been thoroughly demonstrated as an effective and convenient approach in industrial production. In this study, we employed a high-throughput screening strategy to identify a 5′ UTR sequence from the genome of *B. subtilis* 168. This sequence resulted in a 5.8-fold increase in the expression level of EGFP. By utilizing the 5′ UTR sequence to overexpress individual genes within the *rib* operon, it was determined that the genes *ribD* and *ribAB* serve as rate-limiting enzymes in the riboflavin synthesis pathway. Constructing a 5′ UTR library to regulate EGFP expression resulted in a variation range in gene expression levels exceeding 100-fold. Employing the same 5′ UTR library to regulate the expression of EGFP and mCherry within the operon led to a change in the expression ratio of these two genes by over 10,000-fold. So, employing a 5′ UTR library to modulate the expression of the *rib* operon gene and construct a synthetic *rib* operon resulted in a 2.09-fold increase in riboflavin production. These results indicate that the 5′ UTR sequence identified and characterized in this study can serve as a versatile synthetic biology toolkit for achieving complex metabolic network reconstruction. This toolkit can facilitate the fine-tuning of gene expression to produce target products.

## 1. Introduction

Synthetic biology, an emerging field, concentrates on engineering approaches to design, construct, and characterize biological systems, encompassing tools, modular DNA, and computational models [1]. Currently, the primary challenge lies in controlling the engineering pathway or circuit of gene expression within a specific organism. Precisely controlling gene expression by editing the base sequences of coding and non-coding regions has garnered significant research interest in recent years [2,3,4]. Gene expression regulation is mediated by protein abundance and manifested through cell behavior. Protein abundance is correlated with the mRNA level, translation rate, and degradation rate, which are primarily determined by factors such as the promoter, 5′-untranslated region (5′ UTR), ribosomal binding site (RBS), mRNA secondary structure, codon usage, ribosome binding, and terminator sequence [5,6,7]. In *E. coli*, synthetic promoters constructed via promoter engineering exhibit diverse gene expression levels, dynamic ranges, and ligand sensitivities. These synthetic promoters typically demonstrate activity levels that are 50- to 100-fold higher than those of natural promoters [8]. Guiziou et al. developed a gene toolbox comprising libraries of promoters, RBSs, and protein degradation tags to finely control gene expression in *B. subtilis*, achieving the regulation of the GFP concentration within a range of five orders of magnitude. This study significantly broadened the repertoire of regulatory elements available for *B. subtilis* [9]. While the fundamental factors influencing gene transcription and translation efficiency in prokaryotes have received widespread attention, achieving precise control over these processes and their application in metabolic engineering remain subjects of active research [10,11].

The 5′-untranslated region (5′ UTR) of prokaryotic mRNA plays a central role in controlling translation initiation and post-transcriptional regulation by interacting with ribosomes [12,13,14]. It regulates protein expression by controlling mRNA stability and ribosome binding efficiency, enabling temporary translation regulation in response to changes in growth conditions or specific needs for rapid adaptation [15,16,17]. In bacteria and archaea, translation initiation typically commences with the binding of the small 30S ribosomal subunit to the ribosome binding site (RBS) within the 5′ UTR of mRNA [18,19,20]. Various mRNA structures may form before translation initiation, regulating gene expression through transiently stalled ribosomes [15,21]. Hence, considerable research has been undertaken to elucidate the correlation between 5′ UTR sequences and protein expression [22,23,24]. Xiao et al. developed a biophysical model based on the principles of thermodynamics and a four-parameter free energy model to predict the ribosomal translation initiation rate of synthetic 5′ UTRs with large structures, diverse shapes, and multiple spare site modules. The optimization of the 5′ UTR sequence can increase EGFP expression by 50-fold in *B. licheniformis* DW29 [12]. Dvir et al. analyzed the impact of 5′ UTR sequences on yeast protein levels by constructing a comprehensive 5′-UTR library. Their findings revealed that mutations within 1–10 nucleotides upstream of the start codon led to protein level variations of up to seven-fold across the library [6]. In the development of phenyllactic acid engineering strains, Jia et al. established a library combining promoters and 5′ UTRs (PUTRs) to achieve high and consistent protein expression levels. This approach led to a significant 79.2% enhancement in phenyllactic acid production [25]. Therefore, the 5′ UTR sequence plays a pivotal role in augmenting protein expression and regulating metabolic flux in the field of metabolic engineering.

To precisely control target gene expression and balance metabolic fluxes toward the target product, sequence libraries are commonly employed to modulate the gene expression levels and thresholds of gene circuits. Promoters and ribosome binding sites (RBSs) are frequently utilized to enhance gene expression to achieve the maximum formation of desired products. In Gram-positive bacteria, a library comprising 24 constitutive promoters was established, exhibiting an activity range of 380-fold in Staphylococcus aureus and 122-fold in *B. subtilis* [26]. In the production of bacteriocins, incorporating a robust ribosomal binding sequence into the 5′-UTR of the *B. subtilis* bac operon led to a 2.87-fold increase in bacteriocin production [27]. Liu et al. developed a synthetic promoter library in *B. subtilis* aimed at modulating metabolic pathways to enhance inosine and acetyl sugar production, resulting in 700% and 44% increases, respectively, compared to the control strain [28]. In *E. coli*, constructing libraries of promoters and/or RBSs and selecting suitable promoters or RBSs have proven effective in optimizing the PHA synthesis pathway to enhance PHA production [29]. To further enhance translation efficiency, Zhang et al. devised a novel mRNA leader sequence incorporating multiple RBSs, significantly enhancing the translation efficiency in the Gram-positive industrial strain *Bacillus licheniformis* [30]. However, synthesizing target products, particularly natural ones, often requires coordination among multiple genes and metabolic pathways, posing a challenge in metabolic engineering.

In metabolic engineering, coordinating the expressions of multiple genes and elements at both the transcriptional and translational levels is the primary strategy for enhancing target product yields. In general, promoter engineering and RBS engineering are common and clear strategies for fine-tuning protein expression in eukaryotes and prokaryotes [9,28,31]. In *E. coli*, highly variable simple repetitive sequences are utilized to create expression libraries that are capable of enhancing and predicting sample gene expression levels up to 1000-fold [4]. Xu et al. developed a yeast cell promoter library to modulate the expression of green fluorescent protein (GFP) over a 500-fold range. Subsequently, they utilized this chimeric promoter library to enhance squalene production by 10-fold [32]. Coupling the promoter with an RBS library to fine-tune gene expression expanded the regulatory range to four orders of magnitude. Employing this strategy to fine-tune the gene clusters within *Corynebacterium glutamicum*’s arginine synthesis pathway resulted in a 1.61-fold increase in arginine production and a 2.35-fold increase in citrulline production [33]. Additionally, an expression module based on promoters, RBSs, and terminator libraries was engineered through combinatorial methods, achieving a regulatory range of gene expression spanning 627-fold [34]. Most previous studies on gene expression tuning have primarily concentrated on a few model microorganisms, like *E. coli* and *Staphylococcus aureus*. Meanwhile, other important industrial hosts, such as *B. subtilis*, have received scant attention.

*B. subtilis* is acknowledged as a safe microorganism and holds extensive application in industrial production [35]. In this study, a high-throughput screening method was used to screen a new 5′ UTR sequence from the *B. subtilis* genome, which can increase EGFP expression levels by 5.8-fold. To expand the toolbox of *B. subtilis*, a 5′ UTR library was constructed to coordinate gene expression, which modulated the range of EGFP expression levels by over 100-fold. The UTR4 library demonstrates robust fine-tuning capability when regulating multiple genes within a gene cluster, resulting in a range of expression ratios of double reporter genes within the operon exceeding 10,000-fold. By using the 5′ UTR library to construct an artificial *rib* operon, riboflavin production was increased by 2.09-fold. The results suggest that the 5′ UTR library is a potent tool for precisely adjusting the expression of multiple genes within gene clusters, thereby broadening the repertoire of genetic regulatory tools available for fine-tuning gene expression in *B. subtilis.*

## 2. Materials and Methods

### 2.1. Strains, Plasmids, and Culture Conditions

The strains used in this study are listed in Table 1. *E. coli* strain JM109 and *B. subtilis* 168 were cultured at 37 °C in LB media (10 g/L tryptone, 5 g/L yeast extract, and 10 g/L sodium chloride). Plasmids were cloned using *E. coli* JM109 as the host. Strains were cryopreserved at −80 °C for long-term storage until further use. Before use, they were activated on LB agar plates and incubated at 37 °C. When required, the medium was supplemented with specific antibiotics to maintain plasmid stability and prevent contamination. The concentrations used were ampicillin at 100 μg/mL and kanamycin at 50 μg/mL.

### 2.2. Molecular Biology

All of the primers used in this study are listed in Table S1. Amplification of genes was carried out on the *rib* operon using the *B. subtilis* 168 genome as a template. The amplified PCR fragments were joined by fusion PCR to construct recombinant plasmids. PCR amplification was carried out using 2 × Phanta Max Master Mix P515 (Vazyme Biotech Co., Ltd., Nanjing, China) with primers purchased from Azenta (Suzhou, China). Then, the fusion PCR fragment was inserted into the plasmid pP43NMK by Gibson assembly to construct the target plasmid. The transcription of the reporter operon was controlled by the strong promoter P_43_. The ligation products were transformed into competent *E. coli* JM109 cells, and the transformants were screened with ampicillin. In order to ensure the saturation of the library, *E. coli* cells were scraped from three LB solid plates to extract plasmids, and then the plasmids were transformed into *B. subtilis* cells to construct the *B. subtilis* library. Plasmid extraction and DNA purification were performed using the Plasmid Mini Kit and Gel DNA Extraction Mini Kit (TIANGEN, Beijing, China), respectively, following the manufacturer’s recommended protocols. 

### 2.3. Screening and Characterization of 5′ UTR

The genome of *B. subtilis* 168 was isolated using a Bacteria DNA Isolation Mini Kit (TIANGEN, Beijing, China) according to the manufacturer’s instructions and stored for future use. Fragmentation of the *B. subtilis* 168 genome was achieved using an ultrasonic cell disruptor (SCIENTZ, Ningbo, China) with a total power of 750 W. Adjustment of disruption power and time controlled the resulting genome fragments to range between 50 and 300 bp. Post-fragmentation, DNA fragments were combined with an equal volume of 2× absolute ethanol and chilled at −20 °C for 2 h to facilitate DNA precipitation. Samples were then centrifuged at 12,000 rpm for 10 min to pellet the DNA fragments, followed by air-drying at room temperature. Upon drying, DNA fragments were dissolved in sterile water and stored at −20 °C until further use. The Klenow enzyme was utilized to blunt the end of the DNA fragment, and then it was ligated to the plasmid pP43NMK-egfp, which was linearized using P43NMK-library-F/P43NMK-library-R primers. The resulting product was transformed into *E. coli* JM109 to obtain a DNA library. To create a *B. subtilis* library, 10 μg of plasmid DNA from the *E. coli* library was introduced into 10 mL of *B. subtilis* 168 competent cells. The transformed cells were spread onto kanamycin-containing plates at a concentration of 50 μg/mL per plate. Following a 12 h incubation period, colonies were harvested from the plates using LB medium and washed thrice with sterile PBS. The library was sorted using a flow cytometer, and the sorted cells were then plated onto LB agar plates. After 24 h of incubation, colonies showing high fluorescence intensity were picked and inoculated into a 96-well plate. The fluorescence levels of the colonies were subsequently measured using a microplate reader.

### 2.4. Identification of Effective 5′ UTR Sequences

PCR was employed to systematically shorten the 5′ UTR sequence to identify effective sequences within it. Plasmids with varying lengths of the 5′ UTR sequence were constructed using different combinations of primers, namely UTR2-EGFP-F, UTR3-EGFP-F, and UTR4-EGFP-F2, paired with the EGFP-R primer. Then, the PCR fragment was inserted into the pP43NMK plasmid by Gibson assembly to construct the target plasmid. The transcription of the reporter operon was controlled by the strong promoter P_43_. The ligation products were transformed into competent *E. coli* JM109 cells, and the transformants were screened with ampicillin. Subsequently, these plasmids were introduced into *B. subtilis* 168 to construct strains with 5′ UTRs of varying lengths. The fluorescence intensity of EGFP was measured to evaluate the impact of the 5′ UTR length on gene expression. Recombinant strains containing 5′ UTR sequences of varying lengths were inoculated onto an LB medium and incubated at 37 °C with 220 rpm orbital shaking for 10 h. Subsequently, 1% inoculum was transferred to LB medium containing 200 μL and incubated in 96-well sterile black plates (Corning 3603, New York, NY, USA) at 37 °C for 4 h. Finally, the EGFP fluorescence (excitation at 490 nm; emission at 530 nm) and optical density (absorbance at 600 nm) were measured using a Microplate Multimode Reader (Cytation 3, BioTek, Winooski, VT, USA). The wild-type *B. subtilis* 168 strain was used as the negative control, and its fluorescence intensity was subtracted as background.

### 2.5. Construction of 5′ UTR Library

Random nucleotides were incorporated into the 5′ UTR sequence through PCR to create a diverse library of 5′ UTR sequences. Specifically, within the 19-nucleotide range of the 5′ UTR of the primer, the rule dictates randomly replacing one nucleotide (“N”) every three nucleotides. Four primers, namely dppA-F5N1-GFP-F, dppA-F5N2-GFP-F, dppA-F5N3-GFP-F, and dppA-F5N4-GFP-F, were designed to generate a 5′ UTR library. As a result, four separate 5′ UTR libraries were generated, each covering distinct nucleotide positions. In summary, this combination library contains mutations at each position of the 5′ UTR sequence. The EGFP-N1-F, EGFP-N2-F, EGFP-N3-F, and EGFP-N4-F primers, along with the EGFP-N-R primer, were utilized to amplify the 5′ UTR-EGFP fragment. Subsequently, the resulting PCR fragments containing the target gene were inserted into the pP43NMK plasmid using Gibson assembly to construct the 5′ UTR library. The constructed library was then transformed into *B. subtilis*, and the fluorescence intensity of the cells was measured. The expression of the target gene was controlled by the strong promoter P_43_.

For the construction of *egfp* and *mCherry* dual reporter gene libraries, the primer design guidelines established for the 5′ UTR library were followed. We used the upstream primers EGFP-N1-F, EGFP-N2-F, EGFP-N3-F, and EGFP-N4-F paired with the downstream primer G-R-R1 to amplify EGFP fragments with a 5′ UTR library incorporated. We combined the resulting four PCR products to form a UTR4-EGFP fragment library. We employed the upstream primers G-R-N1-F2, G-R-N2-F2, G-R-N3-F2, and G-R-N4-F2 with the downstream primer G-R-R2 to introduce the UTR4 library upstream of *mCherry*. We mixed the obtained PCR products to create UTR4-mCherry library fragments. Fusion PCR was employed to merge the UTR4-EGFP library fragment with the UTR4-mCherry library fragment, followed by their connection to the pP43NMK plasmid via Gibson assembly. We transformed the assembled product into *E. coli* JM109 cells and established a plasmid library by screening the transformants with ampicillin. Subsequently, we introduced these plasmid libraries into *B. subtilis* 168 to establish a dual reporter gene library regulated by UTR4. Additionally, we utilized the G-R-F1, G-R-R1, G-R-F2, and G-R-R2 primers to develop control strains.

### 2.6. Flow Cytometer Sorting and Analysis

The *B. subtilis* library containing the 5′ UTR variants was sorted based on EGFP fluorescence intensity. All sorting was performed on Aria II (BD Biosciences, Franklin Lakes, NJ, USA) using the EGFP channel (488 nm excitation laser, 505 nm dichroic, 525/50 nm emission filter). Cells with increased fluorescence intensity were identified by comparison with a negative control strain lacking the EGFP reporter gene and a positive control strain containing the reporter without the 5′ UTR sequence. The flow cytometry method used to analyze the fluorescence diversity of the 5′ UTR library was consistent with the sorting process. The dual-fluorescent 5′ UTR library was assessed using both the EGFP channel and the mCherry channel (561 nm excitation laser, no dichroic, 582/18 nm emission filter). Flowjo_V1 software was used for data acquisition and analysis. The negative control consisted of a strain lacking the reporter plasmid.

### 2.7. Rib Operon Library Construction

The rib operon fragment was amplified from the *B. subtilis* genome using the P43-rib-F and P43-rib-R primers. Subsequently, the amplified fragment was ligated into a linearized pP43NMK plasmid using a Gibson assembly kit, yielding the recombinant plasmid pNMK-rib. The amplification of the *rib* operon fragment was carried out with the P43-rib-F5-F and P43-rib-R primers, followed by insertion into the pP43NMK plasmid, resulting in P43-UTR4-ribDEBAH. Using a similar method, recombinant plasmids P43-ribD-UTR4-ribEBAH, P43-ribDE-UTR4-ribBAH, and P43-ribDEBA-UTR4-ribH were also constructed. Recombinant plasmids P43-UTR4-ribDEBAH, P43-ribD-UTR4-ribEBAH, P43-ribDE-UTR4-ribBAH, and P43-ribDEBA-UTR4-ribH were transformed into *B. subtilis* 168 to obtain recombinant strains BSU1, BSU2, BSU3, and BSU4.

To construct the rib operon library, a mixed primer was used to introduce a 5′ UTR library before the gene. The amplification of the *rib* operon fragment involved a primer mixture comprising ribDEBAH-F1-1, ribDEBAH-F1-2, ribDEBAH-F1-3, and ribDEBAH-F1-4, along with ribDEBAH-R1. Subsequently, the amplified fragment was integrated into the pP43NMK plasmid using Gibson assembly, thereby constructing the P43-UTR-ribDEBAH library. Using the same method, we constructed a P43-ribD-UTR-ribEBAH library using the mixed primers ribDEBAH-F2-1, ribDEBAH-F2-2, ribDEBAH-F2-3, and ribDEBAH-F2-4 with ribDEBAH-R2. We constructed a P43-ribDE-UTR-ribBAH library using the mixed primers ribDEBAH-F3-1, ribDEBAH-F3-2, ribDEBAH-F3-3, and ribDEBAH-F3-4 with ribDEBAH-R3. We constructed a P43-ribDEBA-UTR-ribH library using the mixed primers ribDEBAH-F4-1, ribDEBAH-F4-2, ribDEBAH-F4-3, and ribDEBAH-F4-4 with ribDEBAH-R4. The ligation products were transformed into competent *E. coli* JM109 cells, and the transformants were screened with ampicillin. Subsequently, the plasmids from four libraries were mixed and transformed into *B. subtilis* 168 to create a composite *rib* library. The transcription of the rib operon was controlled by the strong promoter P_43_. The resulting product was transformed into *E. coli* JM109 to obtain a *rib* operon library. Then, the *rib* operon library was transformed into competent cells of *B. subtilis* 168.

### 2.8. Fluorescence Assays

Fluorescence intensities of EGFP and mCherry were determined as previously reported [36]. Single colonies were selected and inoculated into 96-well black plates (Corning 3603), each containing 200 μL of LB medium. The plates were incubated at 37 °C for 10 h. After incubation, the EGFP fluorescence (excitation at 490 nm; emission at 530 nm), mCherry fluorescence (excitation at 588 nm), and optical density (absorbance at 600 nm) were measured using a Microplate Multimode Reader (BioTek, Cytation 3). Relative fluorescence density was calculated using Equation (1), where FP_bg_ represents the fluorescence value of the strain without the fluorescent protein, and OD_bg_ indicates the absorbance of the medium.

(1) $$\left(\frac{Fp}{OD})=(\frac{FP-{FP}_{bg}}{OD-{OD}_{bg}}\right)$$

### 2.9. Cultivation in Shake Flasks

We inoculated individual colonies into 10 mL of LB medium and shook them at 37 °C and 180 rpm. After incubating for 16 h, we transferred the culture to a 250 mL shake flask containing 50 mL of LBG medium (40 g/L glucose, 5 g/L yeast extract, 10 g/L peptone, and 10 g/L sodium chloride) and shook it at 41 °C and 220 rpm for 24 h. The seed cultures were transferred to a 500 mL baffled shake flask containing 50 mL of fermentation medium according to a 3% inoculum volume and incubated at 41 °C and 220 rpm for 48 h. The shake flask fermentation medium consisted of 40 g/L glucose, 20 g/L yeast extract, 4 g/L ammonium citrate, 1 g/L K_2_HPO_4_, 1 g/L KH_2_PO_4_, 2 g/L MgSO_4_·7H_2_O, 0.04 g/L MnCl_2_, 0.06 g/L calcium chloride, 2 g/L CuSO_4_, and pH 6.9.

### 2.10. Analytical Methods

Screening riboflavin mutants based on their fluorescence characteristics involves measuring fluorescence values at excitation wavelengths of 444 nm and absorption wavelengths of 500 nm [36]. Cell growth was monitored by measuring optical density at 600 nm (OD_600_) using a spectrophotometer. To measure riboflavin concentrations, we diluted the sample with 0.01 M of NaOH until it was within the linear range of the spectrophotometer (0.3–0.8), followed by centrifugation at 12,000 rpm for 2 min to eliminate cells. We immediately measured the absorbance of the supernatant at OD_444_ and then determined the concentration of riboflavin using a standard curve of riboflavin concentrations [36,37].

### 2.11. Statistical Analysis

The data were expressed as mean ± standard deviation. Statistical differences in the experimental data were assessed using one-way analysis of variance (ANOVA). Statistical analysis and plots were performed using the software prism7 (version 7.0, GraphPad Software Inc., San Diego, CA, USA).

## 3. Results

### 3.1. Screening and Identification of 5′ UTR

**Figure 1:**
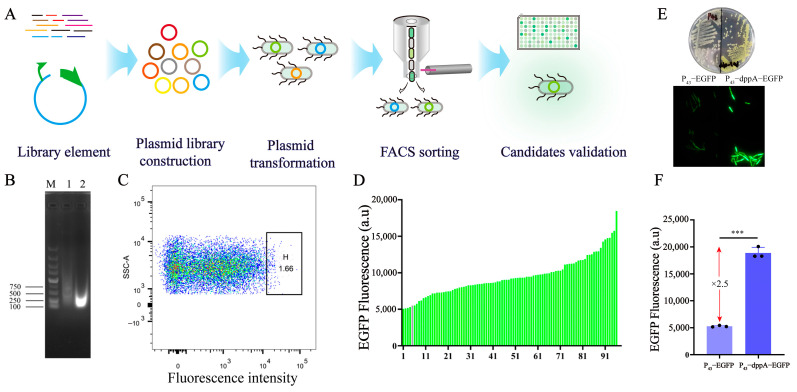
The primary strategy used for screening and identifying 5′ UTR sequences. (**A**) A schematic diagram illustrating the process of 5′ UTR screening. (**B**) Ultrasonication was utilized to disrupt the genome of *B. subtilis* 168. 1: Ultrasonic crushing for 5 s with a power of 10%; 2: ultrasonic crushing for 10 s with a power of 10%. (**C**) Flow cytometry sorting of the highest expressed cell population. (**D**) The determination of fluorescence values was conducted for the strains selected from the library. (**E**) Fluorescence imaging of mutants was carried out using a fluorescence microscope. (**F**) The effect of 5′ UTR sequences on EGFP expression. Black dots represent three duplicate samples. Error bars: ±SD over three independent experiments (*** *p* < 0.001).

Protein synthesis comprises two primary steps: transcription and translation. Translation efficiency is primarily regulated by the characteristics of the 5′ UTR region, governing the recruitment of ribosomes [38]. In order to enrich the synthetic biology toolkit for prokaryotic cells and regulate the expression levels of specific genes, we employed a high-throughput screening strategy to identify novel 5′ UTR sequences (Figure 1A). Stronger 5′ UTR sequences were identified from the genome of *B. subtilis* 168 and applied in the metabolic engineering of *B. subtilis*. Initially, the genome of *B. subtilis* 168 was fragmented into segments ranging from 50 to 300 bp using an ultrasonic disintegrator with a total power of 750 W. The results indicated that the DNA fragments in lane 1 exhibited a diffuse pattern and were distributed across the range of 250 to 1500 bp, with a relatively dispersed distribution. In lane 2, the DNA fragments were predominantly concentrated within the 100–300 bp range, which is consistent with the expected fragment size (Figure 1B). Next, the fragmented genomic DNA was inserted upstream of the reporter gene *egfp* to create a plasmid library comprising random genomic fragments. The plasmid library was then transformed into *B. subtilis* and sorted using flow cytometry. The results suggest that EGFP expression in the library is randomly and uniformly distributed, indicating the presence of 5′ UTR sequences with varying regulatory intensities (Figure 1C). Flow cytometry was employed to sort strains exhibiting higher fluorescence values, comprising 1.66% of the total library population (Figure 1C). The fluorescence intensity of the sorted bacteria was plated on LB plates and re-screened using 96-well plates. The results revealed that 96% of the strains exhibited higher EGFP expression intensity compared to the control strains (Figure 1D). 

The strain with the highest fluorescence intensity was chosen from the library strains as the candidate strain. Subsequently, the candidate strain was streaked on LB plates and incubated for 24 h. The colonies of the candidate strain exhibited a distinct fluorescent yellow color under natural light, indicating a high expression of EGFP (Figure 1E). Fluorescence microscopy revealed that the candidate strain exhibited higher fluorescence intensity in individual cells compared to the control strain (Figure 1E). A further analysis of the fluorescence intensity in the candidate strain revealed a 2.5-fold increase in fluorescence compared to the control strain (Figure 1F). Sequencing of the inserted fragments in the candidate strains unveiled that the random sequence placed upstream of the reporter gene *egfp* was a 128 bp sequence located upstream of the *dppA* gene. The *dppA* gene encodes D-aminopeptidase, which is involved in spore production [39]. In bacteria, the non-coding region upstream of genes plays a crucial role in determining gene expression.

### 3.2. Identification of Effective 5′ UTR Sequences and mRNA Secondary Structure Analysis

**Figure 2:**
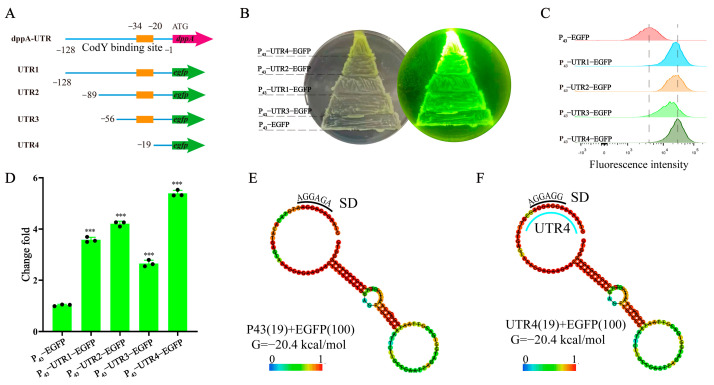
A description of the characteristics and expression ability of the 5′ UTR sequence. (**A**) The features of the 5 ‘UTR sequence. The sequence of P_43_-UTR1 matches that of P_43_-dppA-EGFP. For clarity, the term P_43_-UTR1 will be consistently employed in the subsequent text. (**B**) Fluorescent colony imaging using blue light irradiation. (**C**) A flow cytometry analysis of the effect of different UTR lengths on EGFP expression. (**D**) A measurement of the effect of different UTR lengths on the expression intensity of EGFP. (**E**) The secondary structure of P_43_-EGFP. (**F**) The secondary structure of the UTR4-EGFP sequences. The secondary structure contains 19 bp (5′-GTAAAAAAGGAGGAGCGTT-3′) of UTR4 and 100 bp of EGFP. Black dots represent three duplicate samples. Error bars: ±SD over three independent experiments (*** *p* < 0.001).

A bioinformatics analysis of the 5′ UTR sequence identified the presence of the transcription factor CodY binding sequence (AATATTCATAATTTA) upstream of the *dppA* gene (Figure 2A) [40]. Additionally, to pinpoint effective 5′ UTR sequences and streamline the construction of 5′ UTR libraries for gene expression regulation, shorter 5′ UTR sequences are essential. To expand the search for shorter 5′ UTRs, we identified effective sequences by systematically shortening them. Specifically, we progressively truncated the 5′ UTR upstream of *dppA* through PCR amplification, measuring the fluorescence value to verify its expression intensity (Figure 2A). The shortened UTR sequences are listed in Table S2. The colony color indicated a superior performance of the truncated 5′ UTR sequence, displaying significantly higher fluorescence values compared to those of the control strain (Figure 2B). A flow cytometry analysis revealed that the expression intensity of P_43_-UTR1-EGFP significantly exceeded that of P_43_-EGFP, which is consistent with the enzyme-linked immunosorbent assay results (Figure 1F). Altering the UTR sequence length impacts EGFP expression; UTR4 facilitates the most robust EGFP expression, with UTR2 exhibiting intensity akin to UTR1, whereas UTR3 diminishes EGFP expression intensity (Figure 2C). Further fluorescence measurements demonstrated that UTR4 enhanced the reporter gene expression by 50.53% compared to the original UTR1 sequence. Moreover, the UTR4 sequence led to a 5.8-fold increase in reporter gene expression relative to the control strain (Figure 2D).

Next, we examined whether the secondary structure of mRNA (including 50-UTR and coding region) is correlated with translation efficiency. The RNAfold web server (http://rna.tbi.univie.ac.at//cgi-bin/RNAWebSuite/RNAfold.cgi (accessed on 18 February 2024)) was utilized for the mRNA secondary structure containing UTRs of different lengths and the 100 nt of the *egfp* gene. The predicted mRNA secondary structure showed that the UTR4 sequence did not form any form of secondary structure, showing the same secondary structure as P_43_-EGFP, and both had a Gibson free energy value (Δ*G*) of −20.4 kcal/mol (Figure 2E,F). In previous studies, Gibbs free energy has been shown to be an important factor affecting the performance of 5 ‘UTR, but our results are inconsistent with previous reports. These different results may be due to the significant differences in the 5′ UTR sequence in this study. Previous reports indicate that, among these factors, the higher the matching degree between the SD sequence and 16S rDNA (5′-UCCUCC-3’), the better the expression performance of the 5′ UTR sequence on GFP [41]. The matching degree between the SD sequence in UTR4 and 16SrRNA is higher than that of the SD sequence in P_43_-EGFP. Therefore, we speculate that the promotion of gene expression by UTR4 is related to the SD sequence. Furthermore, the Gibbs free energies of UTR1, UTR2, and UTR3 increased gradually, but UTR3 exhibited the weakest promotion of EGFP expression. Notably, even when the SD sequence of UTR2 was entirely concealed within the secondary structure, its expression intensity surpassed that of UTR3 and approached that of UTR1 (Figure S1). Consequently, these 5′-UTRs demonstrated no correlation between secondary structure formation and translation efficiency [42]. These results indicate that the translation efficiency of mRNA may be determined by a combination of factors, such as the sequence of 5′ UTR, secondary structure, and translation initiation efficiency.

### 3.3. UTR4 Was Used to Increase rib Operon Gene Expression

**Figure 3:**
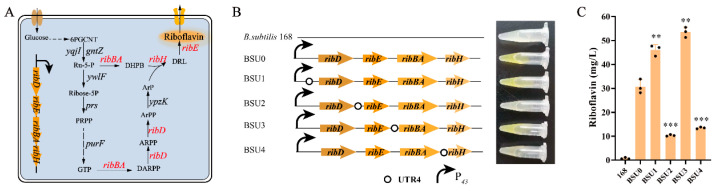
The effect of the overexpression of genes on *rib* operon on riboflavin synthesis. (**A**) The riboflavin synthesis pathway in *B. subtilis*. (**B**) A schematic diagram of genes overexpressing the *rib* operon. (**C**) The determination of the effect of overexpressing genes on riboflavin synthesis on the *rib* operon. Black dots represent three duplicate samples. Error bars: ±SD over three independent experiments (** *p* < 0.01; *** *p* < 0.001).

To investigate the impact of the 5′ UTR sequence on gene expression, we chose the riboflavin synthesis operon *ribDEBAH* (*rib* operon) to assess the expression intensity of the 5′ UTR sequence. The *rib* operon is responsible for the key pathway of riboflavin synthesis (Figure 3A). UTR4 sequences were incorporated upstream of the four genes in the *rib* operon to boost the expression of individual genes with the aims of pinpointing the rate-limiting enzymes in the riboflavin synthesis pathway and alleviating the bottlenecks in this pathway. The fermentation broth of strain BSU0 turned yellow, indicating a significant increase in the riboflavin yield due to *rib* operon overexpression (Figure 3B). The shake flask fermentation results demonstrated that the riboflavin yield rose from 0 mg/L to 30.7 mg/L in *B. subtilis* 168 (Figure 3C). The fermentation broth of strains BSU1 and BSU3 exhibited a yellow hue, indicating riboflavin production. Strain BSU1 yielded 47.7 mg/L of riboflavin, while strain BSU3 yielded 51.3 mg/L. Compared to the control strain BSU0 lacking the 5′ UTR sequence, the riboflavin yield of strain BSU1 increased by 50.16%, suggesting a significant enhancement in riboflavin synthesis with an increased expression of the *ribD* gene. The riboflavin yield of strain BSU3 rose by 74.27%, marking the highest yield among the engineered strains (Figure 3C). In strain BSU3, UTR4 regulates the expression of the gene *ribBA*, suggesting that the enzyme encoded by *ribBA* acts as the rate-limiting factor in riboflavin synthesis, which is consistent with prior findings [43]. The fermentation broth of strains BSU2 and BSU4 exhibited noticeably lighter colors, with riboflavin yields of 10.1 and 13.1 mg/L, respectively, which are significantly lower than that of the control strain BSU0. It is hypothesized that the 5′ UTR may enhance the expressions of the *ribE* and *ribH* genes, leading to a metabolic flux mismatch and thereby inhibiting riboflavin synthesis.

### 3.4. Design and Construction of UTR4 Library

**Figure 4:**
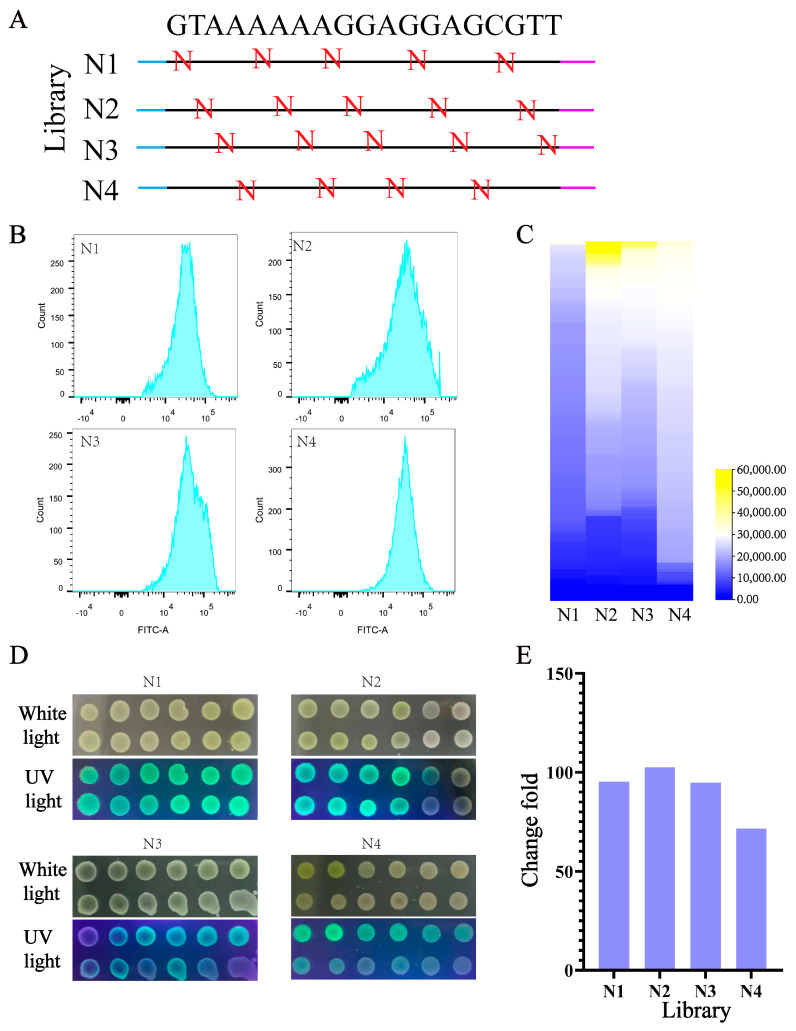
Characterizing the ability of the UTR4 library to regulate gene expression in *B. subtilis*. (**A**) A flow cytometry analysis of the fluorescence value range of four libraries. (**B**) The determination of fluorescence values for mutants in the library was performed. (**C**) Fluorescent colony imaging using white light irradiation and ultraviolet (UV) irradiation. (**D**) The fluorescence value change fold of four libraries. (**E**) The expression change fold of the reporter gene EGFP in four libraries.

Fine-tuning gene expression at the translation level is essential for balancing intracellular metabolic flow. Random alterations to the 5′ UTR sequence can impact mRNA stability and protein expression intensity [44]. To fine-tune the gene expression level, primers were utilized to introduce random bases into the 5′ UTR preceding the ATG of the EGFP gene, resulting in the creation of a random library. Within the 19 bp of the 5′ UTR, a random “N” base was incorporated every three bases, yielding four distinct libraries (Figure 4A). In theory, each primer has the potential to generate 1024 variants, and when combined, four primers can yield a total of 3328 variants. A flow cytometry analysis revealed that the fluorescence intensity of the majority of cells in all four libraries ranged between 10^4^ and 10^5^ (Figure 4B). Notably, library N2 displayed a wider range of expression changes, likely attributed to mutations occurring at critical positions within the regulatory framework of gene expression. A total of 115 single colonies were randomly selected from each of the four libraries and cultured in separate wells of a 96-well plate for 12 h. The analysis revealed a significant variation in fluorescence values across the libraries, with library N4 exhibiting the highest fluorescence intensity among the monoclonal strains (Figure 4C). Monoclonal expression in the N1 library exhibited a narrower range of fluorescence, which is consistent with the findings from flow cytometry. Subsequently, the bacterial liquid dot plate experiment confirmed the substantial range of fluorescence value changes across the library (Figure 4D). The fluorescence variation among the four libraries ranged from a minimum of 70-fold to a maximum of 102-fold (Figure 4E). Upon a comprehensive analysis of libraries N1 through N4, it was observed that the fluorescence range surpassed 180-fold (Figure 4E). 

### 3.5. Tuning the Expression of Multiple Genes in Operons Using the UTR4 Library

**Figure 5:**
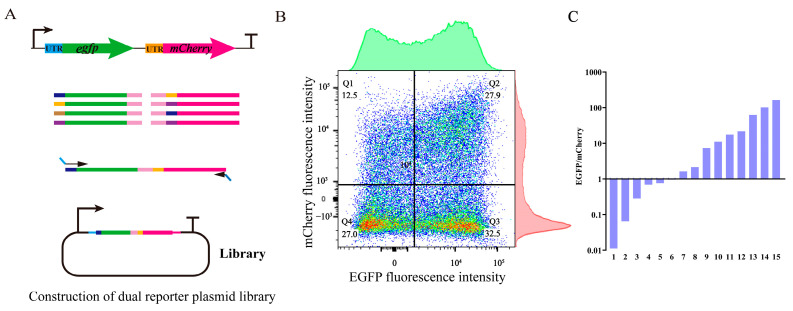
Constructing a UTR4 library to coordinate the expression of dual reporter genes in operons. (**A**) A schematic diagram of the construction of a dual reporter gene library. (**B**) A flow cytometry analysis of the fluorescent protein expression range in dual reporter gene libraries. (**C**) A total of 15 clones were subjected to fluorescence detection during exponential growth to analyze the fluorescence ratio range of EGFP/mCherry.

In metabolic engineering, optimizing gene expression levels is crucial for enhancing yield. Studies have demonstrated that constructing a TIGR library to regulate the expression of two reporter genes within the operon can lead to a 100-fold variation in their relative expression [45]. In a separate investigation, the promoter and downstream 5′-UTR genes of the cluster encoding 1-deoxynojirimycin synthase were optimized, resulting in a final strain yielding 478.62 mg/L of 1-deoxynojirimycin [46]. In this study, the regulatory ability of the UTR4 library to coordinate gene expression within the operon was evaluated by constructing an operon containing dual reporter genes, *egfp* and *mCherry* (Figure 5A). The analysis of the library using flow cytometry revealed that the 5′ UTR effectively coordinated the expression of two reporter genes across a broad spectrum, exhibiting fluorescence intensity ranging from 0 to 10^5^ (a.u.) (Figure 5B). A further analysis revealed that the cells in the library were evenly distributed. Negative cells and double fluorescent cells accounted for approximately the same proportion of the total cell count, representing 27% and 27.9% of the total, respectively. The proportion of cells exhibiting strong EGFP expression was 32.5%, while the proportion of those showing strong mCherry expression was 12.5%, suggesting a higher prevalence of strong EGFP expression (Figure 5B). A total of 15 clones were selected from the library to determine the fluorescence ratio of EGFP to mCherry. The findings revealed a higher number of strains exhibiting robust EGFP expression, aligning with the outcomes obtained through flow cytometry analysis. The ratio of fluorescence values of fluorescent reporter protein EGFP and mCherry varies by more than 10,000-fold (Figure 5C). Hence, employing UTR4 sequence libraries to control the expression of multiple genes within operons holds significant potential in the field of synthetic biology.

### 3.6. The UTR4 Library Was Utilized to Regulate the Expression of Native rib Operon Genes and Construct Synthetic Operons

**Figure 6:**
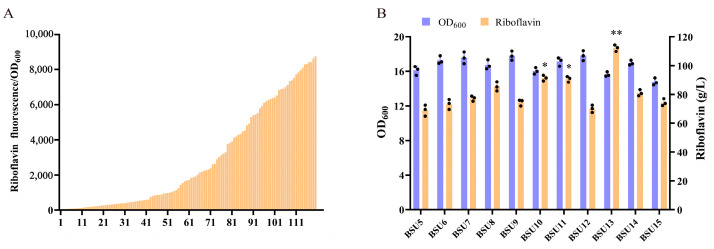
Screening and analysis of *rib* operon library. (**A**) Screening of *rib* operon library based on riboflavin fluorescence values. (**B**) Shake flask fermentation analysis of riboflavin production from 10 mutant strains selected from *rib* operon library. Black dots represent three duplicate samples. Error bars: ±SD over three independent experiments (* *p* < 0.05; ** *p* < 0.01).

Constructing efficient microbial cell factories for natural product biosynthesis necessitates fine-tuning gene expression to optimize the utilization of intermediate products. Employing UTR4 to enhance the expression of each gene in the *rib* operon elucidates the presence of a rate-limiting enzyme within the operon, thereby showcasing its capacity to synthesize the target product. To alleviate the impact of rate-limiting enzymes within the operon and enhance the utilization of intermediate products in the riboflavin synthesis pathway, we utilized a UTR4 library to finely adjust the expression of *rib* operon genes and develop an efficient artificial synthetic operon. Based on the fluorescent properties of riboflavin, we conducted high-throughput screening of artificially synthesized operon libraries using a 96-well plate. We randomly selected 120 artificially synthesized *rib* operons from the library and measured their fluorescence values. The results unveiled substantial variations in fluorescence values, exceeding 1000-fold, underscoring the diversity inherent in the artificially synthesized *rib* library (Figure 6A). As a control, we constructed strain BSU5 by inserting a 5′ UTR sequence upstream of each gene in the rib operon. The results of the shake flask fermentation process revealed that strain BSU13 exhibited the most notable increase in riboflavin production, reaching a maximum yield of 112.2 mg/L. This value represents a 2.09-fold increase compared to the riboflavin production of strain BSU3 (Figure 6B). This suggests that the optimized artificial *rib* operon substantially enhances riboflavin production. Strain BSU12 exhibited the lowest riboflavin production, which did not differ significantly from that of the control strain. However, this strain displayed the highest biomass, potentially competing with riboflavin synthesis for cellular resources.

## 4. Discussion

Controlled gene expression is fundamental to the study of gene function and the ability to modify microorganisms. For example, promoter engineering is a useful tool for the precise control and regulation of gene expression at the transcriptional level and for the modification of ribosome binding sites (RBS), 5′-untranslated regions (5′-UTRs), and RNase III cleavage of mRNA transcripts regulate gene expression at the translational level [47]. However, there is a lack of abundant gene regulatory tools in *B. subtilis*. In this study, a high-throughput screening strategy was used to screen a strong 5′ UTR sequence from the *B. subtilis* genome, which increased the expression level of EGFP by 5.8-fold in *B. subtilis*. The 5′ untranslated region (5′ UTR) is the mRNA structural region upstream of the open reading frame (uORF) and contains ribosome-binding sites, microRNA-binding sites, and structural components involved in the regulation of mRNA stability, pre-mRNA splicing, and translation initiation, and it is critical in determining post-transcriptional control [48]. In previous reports, native PUTR from *E. coli* was screened and engineered, resulting in a 4-fold increase in the translation level compared to P_BAD_ [49]. In another report, the protein expression level was increased by 60-fold by cloning an efficient 5′-mRNA sequence in front of the target gene [50]. In yeast, sequences at positions -1 to -15 upstream of genes have a significant effect on gene expression [51]. In plants, similar results indicate that A residues located at positions −1 to −4 of the 5′ UTR are crucial for high translation efficiency. In plants, similar results indicate that the position of −1 to −4 of the 5′ UTR are crucial for translation efficiency [42]. By progressively shortening the sequence of the 5′ UTR, the 19 bp length proved to have strong translation efficiency, which may be related to the special secondary structure formed by the 5′ UTR. The hairpin structure releases the RBS of the 5′ UTR, allowing for smoother binding of the mRNA to the ribosome, which could be a potential mechanism for high translation levels [49].

Identifying and eliminating rate limiting reactions in synthetic pathways is a key challenge in metabolic engineering and a common and effective metabolic engineering strategy for improving the production of target metabolites. In *E. coli*, fructose diphosphate aldolase (FBA) is the rate-limiting step of glycolysis, and the specific glucose consumption rate of FBA-overexpressing strains is 1.4 times higher than that of control strains [52]. The riboflavin synthesis operon plays a crucial role in the microbial synthesis of riboflavin. By using UTR4 to overexpress each gene on the *rib* operon, it was found that *ribBA* and *ribD* increased the synthesis of riboflavin by 74.27% and 50.16%, indicating that *ribBA* was a rate-limiting step in the riboflavin synthesis pathway. Similar results were reported for a 1.4-fold increase in riboflavin production by the overexpression of *ribBA* [53]. Due to the fact that multiple genes in the operon are controlled by a single promoter, this also leads to the presence of rate-limiting steps in these pathways.

In metabolic engineering, combining multiple related genes into operons is a convenient method to simultaneously regulate multiple genes without the need for multiple promoters. But it also limits the expression regulation of multiple genes within the same operon, leading to the accumulation of intermediate products in the synthesis pathway. Therefore, building an efficient microbial cell factory requires fine-tuning gene expression to maximize the utilization of intermediate products, reduce the accumulation of toxic metabolites, and reduce competition between cell growth and product generation [7]. In order to fine-tune the expression of multiple genes in operons, RBS libraries, 5′ UTR libraries, 3’UTR libraries, and TIRG libraries have been developed. Tunable inter-gene libraries (TIGRs) can alter the relative expressions of two reporter genes within a 100-fold range and balance the expressions of three genes in one operon [45]. In this study, the expression level of EGFP regulated by the UTR4 library changed by more than 100-fold. The UTR4 library was used to regulate the expression of two reporter genes, *egfp* and *mCherry*, within an operon, and the results in a fluorescence range of EGFP/mCherry exceed 10,000-fold. Liu et al. used a synthetic promoter library to produce inosine and acetyl sugar by inhibiting and overexpressing related metabolic pathways, resulting in increases of 700% and 44% in production compared to the control strain [28]. A UTR4 library was inserted into the *rib* operon, and a synthesized *rib* operon was constructed, which increased riboflavin production by 2.09-fold. In metabolic engineering, employing synthetic biology tools to balance the expressions of genes in operons is effective for reducing intermediate product toxicity, enhancing utilization efficiency, and increasing target product yield.

## 5. Conclusions

In conclusion, a 5′ UTR sequence enhancing gene expression in *B. subtilis* was screened and identified. Constructing a library with this 5′ UTR sequence to regulate the expression of either a single gene or multiple genes within an operon can lead to a wide range of changes in gene expression levels. Finally, by utilizing the 5′ UTR library, a synthesized *rib* operon was constructed, leading to a significant increase in riboflavin production.

## Figures and Tables

**Table 1:** The strains and plasmids used in this study.

Strains/Plasmids	Characteristics	Source
Strains		
*B. subtilis* 168	*trpC2*	This lab
*E. coli* JM109	The cloning host, *recA1*, *endA1*, *thi*, *gyr^A96^*, *sup*^E44^, *hsdR*^17^∆ (*lac*-*proAB*)/F′ [*traD36*, *proAB*^+^, *lacI^q^*, *lacZ*∆ M15]	This lab
BSU0	*B. subtilis* 168 derivate, P*_43_*-*rib*	This study
BSU1	BSU0 derivate, *ribD* under the control of UTR4 via the plasmid pP43NMK	This study
BSU2	BSU0 derivate, *ribE* under the control of UTR4 via the plasmid pP43NMK	This study
BSU3	BSU0 derivate, *ribBA* under the control of UTR4 via the plasmid pP43NMK	This study
BSU5	BSU0 derivate, each gene on the *rib* operon under the control of UTR4 via the plasmid pP43NMK	This study
BSU6	*B. subtilis* 168 derivate, *rib* synthesis operator controlled by UTR4 library	This study
BSU7	*B. subtilis* 168 derivate, *rib* synthesis operator controlled by UTR4 library	This study
BSU8	*B. subtilis* 168 derivate, *rib* synthesis operator controlled by UTR4 library	This study
BSU9	*B. subtilis* 168 derivate, *rib* synthesis operator controlled by UTR4 library	This study
BSU10	*B. subtilis* 168 derivate, *rib* synthesis operator controlled by UTR4 library	This study
BSU11	*B. subtilis* 168 derivate, *rib* synthesis operator controlled by UTR4 library	This study
BSU12	*B. subtilis* 168 derivate, *rib* synthesis operator controlled by UTR4 library	This study
BSU13	*B. subtilis* 168 derivate, *rib* synthesis operator controlled by UTR4 library	This study
BSU14	*B. subtilis* 168 derivate, *rib* synthesis operator controlled by UTR4 library	This study
BSU15	*B. subtilis* 168 derivate, *rib* synthesis operator controlled by UTR4 library	This study
Plasmids		
pP43NMK	Amp^r^, Kan^r^, *E. coli*-*B. subtilis* shuttle vector	This lab

## Data Availability

The authors declare that data are contained within the article and in the Supplementary Materials.

## Supplementary Materials

The following supporting information can be downloaded at https://www.mdpi.com/article/10.3390/biology13040277/s1, Figure S1: A description of the secondary structure of the 5′UTR sequence. The black line highlights the SD sequence, while the blue line represents the UTR sequence. (A) The secondary structure of UTR1-EGFP. (B) The secondary structure of UTR2-EGFP. (C) The secondary structure of UTR3-EGFP. Table S1: Primers used in this study. Table S2: Supplementary sequence.

## References

[1] McCarty N.S., Ledesma-Amaro R. (2019). Synthetic Biology Tools to Engineer Microbial Communities for Biotechnology. Trends Biotechnol..

[2] Quax T.E., Claassens N.J., Söll D., van der Oost J. (2015). Codon Bias as a Means to Fine-Tune Gene Expression. Mol. Cell.

[3] Espah Borujeni A., Cetnar D., Farasat I., Smith A., Lundgren N., Salis H.M. (2017). Precise quantification of translation inhibition by mRNA structures that overlap with the ribosomal footprint in N-terminal coding sequences. Nucleic Acids Res..

[4] Egbert R.G., Klavins E. (2012). Fine-tuning gene networks using simple sequence repeats. Proc. Natl. Acad. Sci. USA.

[5] Bentele K., Saffert P., Rauscher R., Ignatova Z., Blüthgen N. (2013). Efficient translation initiation dictates codon usage at gene start. Mol. Syst. Biol..

[6] Mao Y., Liu H., Liu Y., Tao S. (2014). Deciphering the rules by which dynamics of mRNA secondary structure affect translation efficiency in *Saccharomyces cerevisiae*. Nucleic Acids Res..

[7] Li C., Jiang T., Li M., Zou Y., Yan Y. (2022). Fine-tuning gene expression for improved biosynthesis of natural products: From transcriptional to post-translational regulation. Biotechnol. Adv..

[8] Liu X., Gupta S.T.P., Bhimsaria D., Reed J.L., Rodríguez-Martínez J.A., Ansari A.Z., Raman S. (2019). De novo design of programmable inducible promoters. Nucleic Acids Res..

[9] Guiziou S., Sauveplane V., Chang H.J., Clerté C., Declerck N., Jules M., Bonnet J. (2016). A part toolbox to tune genetic expression in *Bacillus subtilis*. Nucleic Acids Res..

[10] Gingold H., Pilpel Y. (2011). Determinants of translation efficiency and accuracy. Mol. Syst. Biol..

[11] Kudla G., Murray A.W., Tollervey D., Plotkin J.B. (2009). Coding-sequence determinants of gene expression in *Escherichia coli*. Science.

[12] Xiao J., Peng B., Su Z., Liu A., Hu Y., Nomura C.T., Chen S., Wang Q. (2020). Facilitating Protein Expression with Portable 5′-UTR Secondary Structures in *Bacillus licheniformis*. ACS Synth. Biol..

[13] Espah Borujeni A., Channarasappa A.S., Salis H.M. (2014). Translation rate is controlled by coupled trade-offs between site accessibility, selective RNA unfolding and sliding at upstream standby sites. Nucleic Acids Res..

[14] Braun F., Durand S., Condon C. (2017). Initiating ribosomes and a 5′/3’-UTR interaction control ribonuclease action to tightly couple *B. subtilis* hbs mRNA stability with translation. Nucleic Acids Res..

[15] Marzi S., Myasnikov A.G., Serganov A., Ehresmann C., Romby P., Yusupov M., Klaholz B.P. (2007). Structured mRNAs regulate translation initiation by binding to the platform of the ribosome. Cell.

[16] Viegas S.C., Apura P., Martínez-García E., de Lorenzo V., Arraiano C.M. (2018). Modulating Heterologous Gene Expression with Portable mRNA-Stabilizing 5′-UTR Sequences. ACS Synth. Biol..

[17] Wen J., Harp J.R., Fozo E.M. (2017). The 5′ UTR of the type I toxin ZorO can both inhibit and enhance translation. Nucleic Acids Res..

[18] Wen J.D., Kuo S.T., Chou H.D. (2021). The diversity of Shine-Dalgarno sequences sheds light on the evolution of translation initiation. RNA Biol..

[19] Simonetti A., Marzi S., Myasnikov A.G., Fabbretti A., Yusupov M., Gualerzi C.O., Klaholz B.P. (2008). Structure of the 30S translation initiation complex. Nature.

[20] Jenner L., Romby P., Rees B., Schulze-Briese C., Springer M., Ehresmann C., Ehresmann B., Moras D., Yusupova G., Yusupov M. (2005). Translational operator of mRNA on the ribosome: How repressor proteins exclude ribosome binding. Science.

[21] Tucker B.J., Breaker R.R. (2005). Riboswitches as versatile gene control elements. Curr. Opin. Struct. Biol..

[22] Salis H.M. (2011). The ribosome binding site calculator. Methods Enzymol..

[23] Petersen S.D., Zhang J., Lee J.S., Jakociunas T., Grav L.M., Kildegaard H.F., Keasling J.D., Jensen M.K. (2018). Modular 5′-UTR hexamers for context-independent tuning of protein expression in eukaryotes. Nucleic Acids Res..

[24] Simonetti A., Marzi S., Jenner L., Myasnikov A., Romby P., Yusupova G., Klaholz B.P., Yusupov M. (2009). A structural view of translation initiation in bacteria. Cell. Mol. Life Sci..

[25] Jia Y., Huang C., Mao Y., Zhou S., Deng Y. (2023). Screening and Constructing a Library of Promoter-5′-UTR Complexes with Gradient Strength in *Pediococcus acidilactici*. ACS Synth. Biol..

[26] Rondthaler S.N., Sarker B., Howitz N., Shah I., Andrews L.B. (2024). Toolbox of Characterized Genetic Parts for *Staphylococcus aureus*. ACS Synth. Biol..

[27] Abdulmalek H.W., Yazgan-Karataş A. (2023). Improvement of Bacilysin Production in *Bacillus subtilis* by CRISPR/Cas9-Mediated Editing of the 5′-Untranslated Region of the bac Operon. J. Microbiol. Biotechnol..

[28] Liu D., Mao Z., Guo J., Wei L., Ma H., Tang Y., Chen T., Wang Z., Zhao X. (2018). Construction, Model-Based Analysis, and Characterization of a Promoter Library for Fine-Tuned Gene Expression in *Bacillus subtilis*. ACS Synth. Biol..

[29] Zhang X., Lin Y., Wu Q., Wang Y., Chen G.Q. (2020). Synthetic Biology and Genome-Editing Tools for Improving PHA Metabolic Engineering. Trends Biotechnol..

[30] Zhang M., Song J., Xiao J., Jin J., Nomura C.T., Chen S., Wang Q. (2022). Engineered multiple translation initiation sites: A novel tool to enhance protein production in *Bacillus licheniformis* and other industrially relevant bacteria. Nucleic Acids Res..

[31] Kotopka B.J., Smolke C.D. (2020). Model-driven generation of artificial yeast promoters. Nat. Commun..

[32] Xu L., Liu P., Dai Z., Fan F., Zhang X. (2021). Fine-tuning the expression of pathway gene in yeast using a regulatory library formed by fusing a synthetic minimal promoter with different Kozak variants. Microb. Cell Fact..

[33] Duan Y., Zhai W., Liu W., Zhang X., Shi J.S., Zhang X., Xu Z. (2021). Fine-Tuning Multi-Gene Clusters via Well-Characterized Gene Expression Regulatory Elements: Case Study of the Arginine Synthesis Pathway in *C. glutamicum*. ACS Synth. Biol..

[34] Xu K., Tong Y., Li Y., Tao J., Rao S., Li J., Zhou J., Liu S. (2022). Autoinduction Expression Modules for Regulating Gene Expression in *Bacillus subtilis*. ACS Synth. Biol..

[35] Bareia T., Pollak S., Eldar A. (2018). Self-sensing in *Bacillus subtilis* quorum-sensing systems. Nat. Microbiol..

[36] You J., Du Y., Pan X., Zhang X., Yang T., Rao Z. (2022). Increased Production of Riboflavin by Coordinated Expression of Multiple Genes in Operons in *Bacillus subtilis*. ACS Synth. Biol..

[37] Man Z.W., Rao Z.M., Cheng Y.P., Yang T.W., Zhang X., Xu M.J., Xu Z.H. (2014). Enhanced riboflavin production by recombinant *Bacillus subtilis* RF1 through the optimization of agitation speed. World J. Microbiol. Biotechnol..

[38] Ingolia N.T. (2014). Ribosome profiling: New views of translation, from single codons to genome scale. Nat. Rev. Genet..

[39] Remaut H., Bompard-Gilles C., Goffin C., Frère J.M., Van Beeumen J. (2001). Structure of the *Bacillus subtilis* D-aminopeptidase DppA reveals a novel self-compartmentalizing protease. Nat. Struct. Biol..

[40] Belitsky B.R., Sonenshein A.L. (2008). Genetic and biochemical analysis of CodY-binding sites in *Bacillus subtilis*. J. Bacteriol..

[41] Rao Y., Li P., Xie X., Li J., Liao Y., Ma X., Cai D., Chen S. (2021). Construction and Characterization of a Gradient Strength Promoter Library for Fine-Tuned Gene Expression in *Bacillus licheniformis*. ACS Synth. Biol..

[42] Kim Y., Lee G., Jeon E., Sohn E.J., Lee Y., Kang H., Lee D.W., Kim D.H., Hwang I. (2014). The immediate upstream region of the 5′-UTR from the AUG start codon has a pronounced effect on the translational efficiency in *Arabidopsis thaliana*. Nucleic Acids Res..

[43] Hümbelin M., Griesser V., Keller T., Schurter W., Haiker M., Hohmann H.P., Ritz H., Richter G., Bacher A., van Loon A.P.G.M. (1999). GTP cyclohydrolase II and 3,4-dihydroxy-2-butanone 4-phosphate synthase are rate-limiting enzymes in riboflavin synthesis of an industrial *Bacillus subtilis* strain used for riboflavin production. J. Ind. Microbiol. Biotechnol..

[44] Jia L., Mao Y., Ji Q., Dersh D., Yewdell J.W., Qian S.B. (2020). Decoding mRNA translatability and stability from the 5′ UTR. Nat. Struct. Mol. Biol..

[45] Pfleger B.F., Pitera D.J., Smolke C.D., Keasling J.D. (2006). Combinatorial engineering of intergenic regions in operons tunes expression of multiple genes. Nat. Biotechnol..

[46] Li X., Zhang M., Lu Y., Wu N., Chen J., Ji Z., Zhan Y., Ma X., Chen J., Cai D. (2023). Metabolic engineering of *Bacillus amyloliquefaciens* for efficient production of α-glucosidase inhibitor1-deoxynojirimycin. Synth. Syst. Biotechnol..

[47] Jin L.Q., Jin W.R., Ma Z.C., Shen Q., Cai X., Liu Z.Q., Zheng Y.G. (2019). Promoter engineering strategies for the overproduction of valuable metabolites in microbes. Appl. Microbiol. Biotechnol..

[48] Ryczek N., Łyś A., Makałowska I. (2023). The Functional Meaning of 5′UTR in Protein-Coding Genes. Int. J. Mol. Sci..

[49] Zhou S., Ding R., Chen J., Du G., Li H., Zhou J. (2017). Obtaining a Panel of Cascade Promoter-5′-UTR Complexes in *Escherichia coli*. ACS Synth. Biol..

[50] Kucharova V., Skancke J., Brautaset T., Valla S. (2013). Design and optimization of short DNA sequences that can be used as 5′ fusion partners for high-level expression of heterologous genes in *Escherichia coli*. Appl. Environ. Microbiol..

[51] Li J., Liang Q., Song W., Marchisio M.A. (2017). Nucleotides upstream of the Kozak sequence strongly influence gene expression in the yeast *S. cerevisiae*. J. Biol. Eng..

[52] Kitamura S., Shimizu H., Toya Y. (2021). Identification of a rate-limiting step in a metabolic pathway using the kinetic model and in vitro experiment. J. Biosci. Bioeng..

[53] Shi T., Wang Y., Wang Z., Wang G., Liu D., Fu J., Chen T., Zhao X. (2014). Deregulation of purine pathway in *Bacillus subtilis* and its use in riboflavin biosynthesis. Microb. Cell Fact..

