# Supplementary material for: Utilizing 5′ UTR Engineering Enables Fine-Tuning of Multiple Genes within Operons to Balance Metabolic Flux in Bacillus subtilis
Source: Biology (Basel). 2024 Apr 19;13(4):277. doi: 10.3390/biology13040277 (PMC11047901; doi:10.3390/biology13040277)
Supplement: Supplementary file 1 [file biology-13-00277-s001.zip › Supplementary Figure S1.pdf]

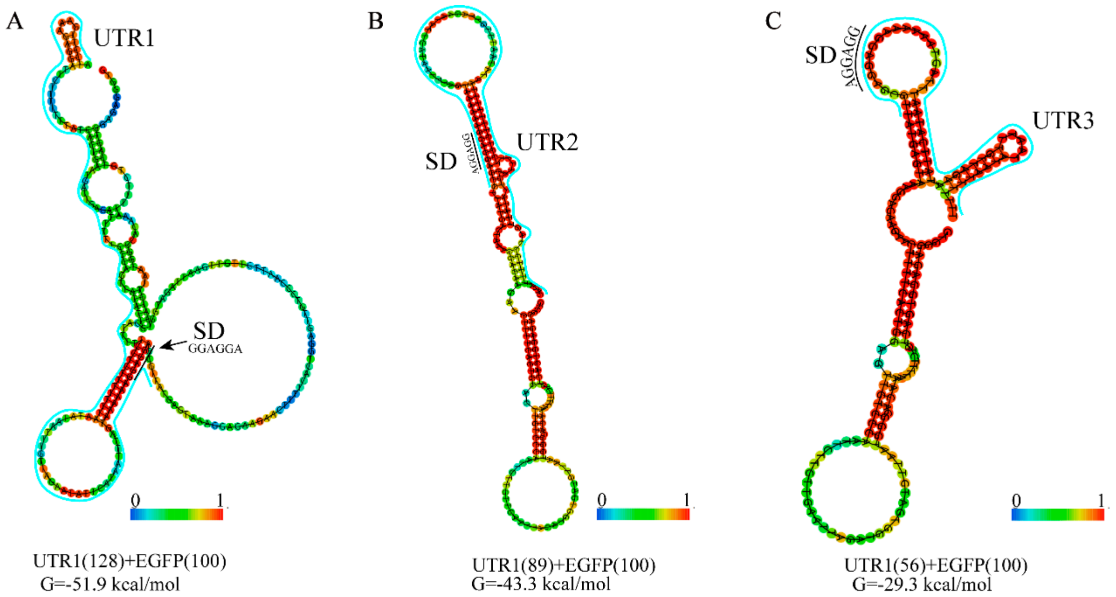

Figure S1. Describe the secondary structure of the 5'UTR sequence. The black line highlights the SD sequence, while the blue line represents the UTR sequence. (A) Secondary structure of UTR1-EGFP. (B) Secondary structure of UTR2-EGFP. (C) Secondary structure of UTR3-EGFP.
