# Supplementary material for: Utilizing 5′ UTR Engineering Enables Fine-Tuning of Multiple Genes within Operons to Balance Metabolic Flux in Bacillus subtilis
Source: Biology (Basel). 2024 Apr 19;13(4):277. doi: 10.3390/biology13040277 (PMC11047901; doi:10.3390/biology13040277)
Supplement: Supplementary file 1 [file biology-13-00277-s001.zip › Table S1.pdf]

Table S1 Primers used in this study.

| **Primer** | **Sequence (5’-3’)** |
|---|---|
| P43NMK- library-F | TGATGAAAGCTTGGCGTAATCATGG |
| P43NMK-library-R | GTGTACATTCCTCTCTTACCTATAATGGTACCG |
| UTR2-EGFP-F | GGTAAGAGAGGAATGTACACGAGATTTTTCCCAGTTATATTGCATTTTTCCTC |
| UTR3-EGFP-F | GGTAAGAGAGGAATGTACACTTTTTTTAATATAATTTGTTAGAATATTCATAATTTAG<br>TAAAAAAGGAGGAGCGTTATG |
| UTR4-EGFP-F | GGTAAGAGAGGAATGTACACGTAAAAAAGGAGGAGCGTTATGAGTAAAGGAG |
| EGFP-R | ATTACGCCAAGCTTTCATCACTATTTGTATAGTTCATCCATGCCATGTGTAATCC |
| EGFP-N1-F | GGTAAGAGAGGAATGTACACGTNAAANAGGNGGANCGTNAT<br>GAGTAAAGGAGAAGAACTTTTCACTGGAG |
| EGFP-N2-F | GGTAAGAGAGGAATGTACACGNAAANAAGNAGGNGCGNTAT<br>GAGTAAAGGAGAAGAACTTTTCACTGGAG |
| EGFP-N3-F | GGTAAGAGAGGAATGTACACNTAANAAANGAGNAGCNTTAT<br>GAGTAAAGGAGAAGAACTTTTCACTGGAG |
| EGFP-N4-F | GGTAAGAGAGGAATGTACACGTANAAANGGANGAGNGTTATGAGTAAAGGAGAAGAACTTTTCACTGGAG |
| EGFP-N-R | CTATTTGTATAGTTCATCCATGCCATGTGT |
| G-R-F1 | GGTAAGAGAGGAATGTACACGTAAAAAAGGAGGAGCGTTATGAGTAAAGGAG |

| | |
|---|---|
| G-R-R1 | GTGTACATTCCTCTCTTACCTATTTGTATAGTTCATCCATGCCATGTGTAATCC |
| G-R-F2 | TGGCATGGATGAACTATACAAATAGGTAAGAGAGGAATGTACACGTAAAAAAGGAGGAGCG TTATGGTGAGCAAGGGCGAG |
| G-R-R2 | ATTACGCCAAGCTTTCATCATTACTTGTACAGCTCGTCCATGCC |
| G-R-N1-F2 | TGGCATGGATGAACTATACAAATAGGTAAGAGAGGAATGTACACNTAANAAANGAGNAGCNTT ATGGTGAGCAAGGGCGAG |
| G-R-N2-F2 | TGGCATGGATGAACTATACAAATAGGTAAGAGAGGAATGTACACGNAAANAAGNAGGNGCGNT ATGGTGAGCAAGGGCGAG |
| G-R-N3-F2 | TGGCATGGATGAACTATACAAATAGGTAAGAGAGGAATGTACACGTNAAANAGGNGGANCGTN ATGGTGAGCAAGGGCGAG |
| G-R-N4-F2 | TGGCATGGATGAACTATACAAATAGGTAAGAGAGGAATGTACACGTANAAANGGANGAGNGTT ATGGTGAGCAAGGGCGAG |
| P43NMK-F | TGATGAAAGCTTGGCGTAATCATGG |
| P43NMK-R | GTGTACATTCCTCTCTTACCTATAATGGTACCG |
| P43NMK-F-F | ATGAGTAAAGGAGAAGAACTTTTCACTGGAG |
| P43NMK-F-R | GTGTACATTCCTCTCTTACCTATAATGGTACCG |
| P43-rib-F | GGTAAGAGAGGAATGTACACATGGAAGAGTATTATATGAAGCTGGCCTTAG |
| P43-rib-R | ATTACGCCAAGCTTTCATCATTATTCAAATGAGCGGTTTAAATTTGCCATTTC |

| | |
|---|---|
| P43-rib-F5-F | GGTAAGAGAGGAATGTACACGTAAAAAAGGAGGAGCGTTATGGAAGAGTATTATATGAAGCTGGCCTTAG |
| ribDEBAH-F1-1 | GGTAAGAGAGGAATGTACACNTAANAAANGAGNAGCNTTATGGAAGAGTATTATATGAAGCTGGCCTTAG |
| ribDEBAH-F1-2 | GGTAAGAGAGGAATGTACACGNAAANAAGNAGGNGCGNTATGGAAGAGTATTATATGAAGCTGGCCTTAG |
| ribDEBAH-F1-3 | GGTAAGAGAGGAATGTACACGTNAAANAGGNGGANCGTNATGGAAGAGTATTATATGAAGCTGGCCTTAG |
| ribDEBAH-F1-4 | GGTAAGAGAGGAATGTACACGTANAAANGGANGAGNGTTATGGAAGAGTATTATATGAAGCTGGCCTTAG |
| ribDEBAH-R1 | GTGTACATTCCTCTCTTACCTATTCCTTTGTCGGTTTTGCCGT |
| ribDEBAH-F2-1 | CCGACAAAGGAATAGGTAAGAGAGGAATGTACACNTAANAAANGAGNAGCNTTATGTTTACAGGAATTATC<br>GAAGAAACAGGC |
| ribDEBAH-F2-2 | CCGACAAAGGAATAGGTAAGAGAGGAATGTACACGNAAANAAGNAGGNGCGNTATGTTTACAGGAATTATC<br>GAAGAAACAGGC |
| ribDEBAH-F2-3 | CCGACAAAGGAATAGGTAAGAGAGGAATGTACACGTNAAANAGGNGGANCGTNATGTTTACAGGAATTATC<br>GAAGAAACAGGC |
| ribDEBAH-F2-4 | CCGACAAAGGAATAGGTAAGAGAGGAATGTACACGTANAAANGGANGAGNGTTATGTTTACAGGAATTATC<br>GAAGAAACAGGC |
| ribDEBAH-R2 | GTGTACATTCCTCTCTTACCTAAAAGCCGTTTTCGCTTAAGAAGG |
| ribDEBAH-F3-1 | CTTAAGCGAAAACGGCTTTTAGGTAAGAGAGGAATGTACACNTA<br>ANAAANGAGNAGCNTTATGTTTCATCCGATAGAAGAAGCACTG |
| ribDEBAH-F3-2 | CTTAAGCGAAAACGGCTTTTAGGTAAGAGAGGAATGTACACGNAAANAAGNAGGNGCGNTATGTTTCATCC<br>GATAGAAGAAGCACTG |

| | |
|---|---|
| ribDEBAH-F3-3 | CTTAAGCGAAAACGGCTTTTAGGTAAGAGAGGAATGTACACGTNAAANAGGNGGANCGTNATGTTTCATCCG ATAGAAGAAGCACTG |
| ribDEBAH-F3-4 | CTTAAGCGAAAACGGCTTTTAGGTAAGAGAGGAATGTACACGTANAAANGGANGAGNGTTATGTTTCATCCGA TAGAAGAAGCACTG |
| ribDEBAH-R3 | GTGTACATTCCTCTCTTACTTAGAAATGAAGTAAATGACCTAGCTTGTTCATTTTGG |
| ribDEBAH-F4-1 | ACTTCATTTCTAAGTAAGAGAGGAATGTACACNTAANAAANGAGNAGCNTTATGAATATCATACAAGGAAATT TAGTTGGTACAGG |
| ribDEBAH-F4-2 | ACTTCATTTCTAAGTAAGAGAGGAATGTACACGNAAANAAGNAGGNGCGNTATGAATATCATACAAGGAAATT TAGTTGGTACAGG |
| ribDEBAH-F4-3 | ACTTCATTTCTAAGTAAGAGAGGAATGTACACGTNAAANAGGNGGANCGTNATGAATATCATACAAGGAAATTT AGTTGGTACAGG |
| ribDEBAH-F4-4 | ACTTCATTTCTAAGTAAGAGAGGAATGTACACGTANAAANGGANGAGNGTTATGAATATCATACAAGGAAATTT AGTTGGTACAGG |
| ribDEBAH-F2 | CCGACAAAGGAATAGGTAAGAGAGGAATGTACACGTAAAAAAGGAGGAGCGTTATGTTTACAGGAATTATCGAA GAAACAGGC |
| ribDEBAH-F3 | CTTAAGCGAAAACGGCTTTTAGGTAAGAGAGGAATGTACACGTAAAAAAGGAGGAGCGTTATGTTTCATCCGATA GAAGAAGCACTG |
| ribDEBAH-F4 | ACTTCATTTCTAAGTAAGAGAGGAATGTACACGTAAAAAAGGAGGAGCGTTATGAATATCATACAAGGAAATTTA GTTGGTACAGG |
| ribDEBAH-R4 | ATTACGCCAAGCTTTCATCATTATTCAAATGAGCGGTTTAAATTTGCCATTTC |
