# Supplementary material for: Utilizing 5′ UTR Engineering Enables Fine-Tuning of Multiple Genes within Operons to Balance Metabolic Flux in Bacillus subtilis
Source: Biology (Basel). 2024 Apr 19;13(4):277. doi: 10.3390/biology13040277 (PMC11047901; doi:10.3390/biology13040277)
Supplement: Supplementary file 1 [file biology-13-00277-s001.zip › Table S2.pdf]

Table S2 Supplementary sequence.

UTR	Sequence (5'-3')
UTR1	**ATCCTTGAAAGAGGATTCTTTTTTTATCACTGAATGATTGAGATTTTTCCCAGTTATATTGCATTTTTCCTCTTTTTTTAATATAATTTGTTAGAATATTCATAATTTAGTAAAAAAGGAGGAGCGTT**ATGAGTAAAGGAGAAGAACTTTTCACTGGAGTTGTCCCAATTCTTGTTGAATTAGATGGTGATGTTAATGGGCACAAATTTTCTGTCAGTGGAGAGGGTG
UTR2	**GAGATTTTTCCCAGTTATATTGCATTTTTCCTCTTTTTTTAATATAATTTGTTAGAATATTCATAATTTAGTAAAAAAGGAGGAGCGTT**ATGAGTAAAGGAGAAGAACTTTTCACTGGAGTTGTCCCAATTCTTGTTGAATTAGATGGTGATGTTAATGGGCACAAATTTTCTGTCAGTGGAGAGGGTG
UTR3	**TTTTTTTAATATAATTTGTTAGAATATTCATAATTTAGTAAAAAAGGAGGAGCGTT**ATGAGTAAAGGAGAAGAACTTTTCACTGGAGTTGTCCCAATTCTTGTTGAATTAGATGGTGATGTTAATGGGCACAAATTTTCTGTCAGTGGAGAGGGTG
UTR4	**GTAAAAAAGGAGGAGCGTT**ATGAGTAAAGGAGAAGAACTTTTCACTGGAGTTGTCCCAATTCTTGTTGAATTAGATGGTGATGTTAATGGGCACAAATTTTCTGTCAGTGGAGAGGGTG

Note: The bold font represents the UTR sequence; The nonbold font represents the 100 bp sequence of EGFP
